# Musculoskeletal pain in schoolchildren across puberty: a 3-year follow-up study

**DOI:** 10.1186/s12969-015-0014-z

**Published:** 2015-05-15

**Authors:** Francesca Sperotto, Sara Brachi, Fabio Vittadello, Francesco Zulian

**Affiliations:** Department of Pediatrics, University of Padua, Padua, Italy

**Keywords:** Musculoskeletal pain, Benign joint hypermobility syndrome, Puberty

## Abstract

**Background:**

Chronic Musculoskeletal Pain (MSP) in children can be due to non-inflammatory conditions, such as the benign joint hypermobility syndrome (BJHS) or idiopathic MSP (IMSP). Aim of the study was to evaluate type and persistence of MSP in a cohort of schoolchildren with MSP followed for 3 years, in order to identify the main risk factors.

**Methods:**

Healthy schoolchildren, aged 8–13 years, underwent a general and rheumatologic examination, focusing on presence of chronic MSP, defined as continuous or recurrent pain lasting more than 3 months and heavily interfering with daily life activities, presence of generalized joint hypermobility, the body mass index and the pubertal stage. All symptomatic subjects were re-evaluated 3 years later with the same methods.

**Results:**

Seventy of the 88 symptomatic subjects of the initial cohort of 289 were re-evaluated 3 years later. Of these, 38 (54.3 %) still presented MSP, including 19 with BJHS and 19 with IMSP. Main symptoms were lower limbs arthralgia and myalgia. MSP persisted more in females than in males (*p* = 0.038) and in pubertal rather than pre-pubertal subjects (*p* = 0.022); these subjects recovered significantly more both from BJHS (*p* = 0.004) and IMSP (*p* = 0.016). Gender did not influence the distribution of MSP according to pubertal stage.

**Conclusions:**

Female gender, BJHS and pubertal stage are important risk factors for persistence of MSP. Further studies are needed to evaluate the natural history of MSP towards adulthood and the role of the pubertal age.

## Background

Chronic Musculoskeletal Pain (MSP) represents one of the most frequent causes of pain and sometimes disability in childhood. Since pain influences the patient’s quality of life, its nature and risk factors should be identified and, if possible, controlled [1–4].

MSP in children can be due to various non-inflammatory conditions and often is misdiagnosed or included in the vast category of “unspecified MSP” [2, 5]. One of the most overlooked conditions is the Benign Joint Hypermobility Syndrome (BJHS), which is a clinical entity characterized by generalized joint hypermobility (GJH) associated with MSP [6–10]. When chronic MSP is not referable to known causes, it is defined as Idiopathic MSP (IMSP) [5, 11]. This group of patients should be properly followed in order to identify more specific causes of pain.

Up to now, only a few studies have evaluated the long-term course of MSP [11–15] and none of them has evaluated every subject with a careful clinical examination. In fact, these studies involved large cohorts of subjects and studied them through standardized postal questionnaires, which improve the standardization but lack important information, such as observational measures, MSP differential diagnosis and parents’ opinions. Some studies, moreover, have evaluated the adolescence period, defined by age [12, 15–17], but none of them has considered the specific and clinically-evaluated pubertal stages in the MSP modification over time.

Aim of our study was to evaluate the persistence of osteoarticular symptoms in a cohort of schoolchildren with MSP followed for 3 years and to analyze the main risk factors for its persistence, with particular attention to the role of puberty.

## Methods

### Study population

The initial study was conducted in June 2009 and included schoolchildren of the District of Padua, Italy. The initial sample consisted of 289 pupils, aged 8–13 years (mean 10.6). The epidemiological project, included possible follow-up during time, was approved by the Ethic Committee of the Padua District Health Authority and by the Schools Directors; moreover a consent form, describing purposes and procedures of the study, was distributed to the schoolchildren’s parents. Once both parents gave informed consent and children assented to research, the subject entered the study. These children underwent a general clinical examination, including puberty and body mass index (BMI) evaluation, a rheumatologic exam focused on GJH test. Two hundreds and eight (72 %) were pre-pubertal and 81 (28 %) were pubertal [18, 19]. Eighty-eight of them (30.4 %) reported chronic musculoskeletal pain, including 38 BJHS and 50 IMSP, such as growing pains [20].

This cohort was re-evaluated 3 years later (June 2012) to determine the persistence of MSP and to investigate factors contributing to it. We used the same methods as in 2009, which we will explain in details in the following paragraphs.

Each patient’s family, contacted by phone, was informed about the aim of the study and was invited to take part to it. As in 2009, on the basis of preliminary parent’s information, children with past or present signs of any neurologic, skeletal, metabolic or autoimmune diseases were excluded.

### Clinical assessment

The past medical history from all participants was collected and a team of three pediatricians carried out the physical examination. The clinical assessment consisted of collecting information about family history for MSP conditions, in order to investigate possible familial predisposition to BJHS, growing pains or other forms of IMSP. Family history of autoimmune diseases was also investigated. Patients were asked to refer about presence and sites of chronic MSP in the previous 6 months by using a standardized form. According to the International Association for the Study of Pain, chronic MSP was defined as continuous or recurrent pain lasting more than 3 months and heavily interfering with daily life activities [21]. Information on age, gender and type of sport activities was also collected. We considered sport activities those performed at school or outside at least twice a week and distinguished those with articular overloading, *e.g.* volleyball, basketball, football, rugby, tennis, from those without articular overloading, *e.g.* swimming and cycling.

General physical examination included weight, height, BMI and pubertal stage evaluation. Obesity was defined as a BMI greater than the 95th percentile for age, while overweight was defined as a BMI between the 85th and the 95th percentile for age, plotted on the Italian version of NCHS (National Center for Health Statistics) curves [22]. The pubertal stage was assessed by the presence of secondary signs of pubertal development. For females, puberty was defined by the stage of breast development (Tanner stage ≥ 3) and menarche. For males, puberty was defined in presence of a testicles volume ≥12 ml and presence of pubic and underarm hair [18, 19].

The rheumatologic examination carefully evaluated the musculoskeletal apparatus and mainly focused on the presence of GJH, identified by Beighton score ≥4/9 [6, 23, 24].

### Statistical analysis

Demographic, clinical and laboratory characteristics of patients were analyzed by descriptive statistics. Absolute and relative frequencies have been reported.

Student’s *t* test for independent samples, Pearson’s *X*^2^ and Fisher’s Exact test-or Fisher Freeman-Halton extension test-were used to compare categorical and continuous variables between subgroups. Clinical variables obtained at baseline (2009) and at the last evaluation (2012) were compared by using the Wilcoxon test and the McNemar test. A value of *p* < 0.05 was considered as significant. The analysis was performed using the StatsDirect statistical software (Version 2,7,8 StatsDirect Ltd, Cheshire, UK).

## Results

### Population characteristics

**Fig. 1:**
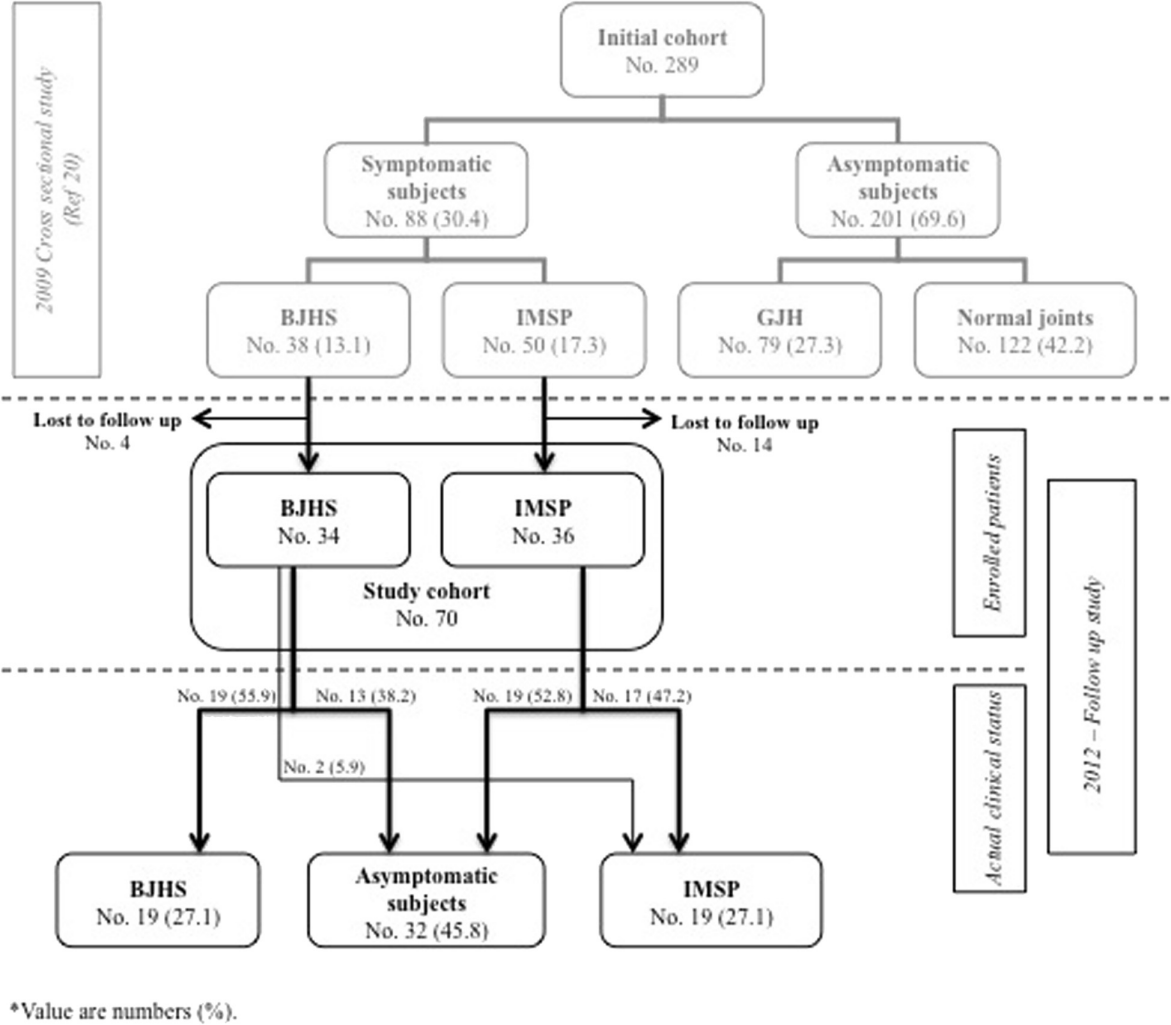
Schematic summary of the study design with relative prevalence of Idiopathic Musculoskeletal Pain and Benign Joint Hypermobility Syndrome at baseline and after 3 year follow-up

Eighty-eight patients with MSP were contacted and 70 entered the follow-up study (Fig. 1). Twenty-nine (41.4 %) were females and 41 (58.6 %) males with a F:M ratio of 1:1,4. The mean age was 14.0 (range 11–16); 68 (97.1 %) subjects were Caucasian, 2 (2.9 %) were non-Caucasian.

On family history, 28 (40.0 %) subjects had at least one first degree relative with BJHS and 5 (7.1 %) with an autoimmune disease.

After 3 year follow-up, 38 subjects (54.3 %) still presented MSP, including 19 with BJHS and 19 with IMSP (Fig. 1). MSP persisted significantly more in females than in males (*p* = 0.038). Interestingly, 13 patients with BJHS were females and 6 males (F:M = 2.2:1) while, among those with IMSP, 7 were females and 12 males (F:M = 0.6:1). In other words, BJHS tends to persist in females while, conversely, IMSP mainly involves males and this difference is close to significance (*p* = 0.051).

Among the 32 subjects who became asymptomatic (45.7 %), 12 (17.1 %) still had various degree of joint hypermobility while 20 (28.6 %) had a normal rheumatologic examination (Fig. 1). Four subjects (5.8 %) were obese but only two were symptomatic.

Main symptoms reported by the symptomatic subjects were arthralgia and myalgia. In general, the most interested body district was the lower limbs (52.6 %), followed by the spine (23.7 %). Diffuse involvement (more than one anatomical district) interested the remaining 23.7 %. Arthralgia was referred by 30/38 (78.9 %) children and mainly involved the lower limbs (63.2 %) and the spine (36.8 %). More rarely, pain interested hips (5.3 %), wrists (5.3 %) and shoulders (2.6 %). Sixteen (42.1 %) subjects presented myalgia, mainly involving the lower limbs. No patient presented enthesopathy, arthritis or fibromyalgia.

As for treatment, 6 subjects (15.8 %) were usually taking non-steroidal anti-inflammatory drugs (NSAIDs) for pain relief.

### MSP persistence and associated variables

The evolution of MSP according to the original subtype, during the 3 years follow-up, is summarized in Fig. 1. IMSP tends to subside more frequently than BJHS over time (52.8 % *vs.* 38.2 % of subjects), but without statistical significance. However, 35.5 % of the patients with BJHS resolved their MSP but remained hypermobile.

Age, BMI and sport were not significantly associated with the persistence of MSP.

To investigate the role of puberty for the persistence of MSP, we stratified the cohort in three groups according to the pubertal stage: the first group (25 subjects) was composed by subjects that were still pre-pubertal; the second included 34 subjects that became pubertal during the 3 years of follow up; group 3 included 11 subjects that were already pubertal in 2009. In general, 25 subjects (group 1, 35.7 %) were pre-pubertal and 45 (groups 2 and 3, 64.3 %) were pubertal.

**Fig. 2:**
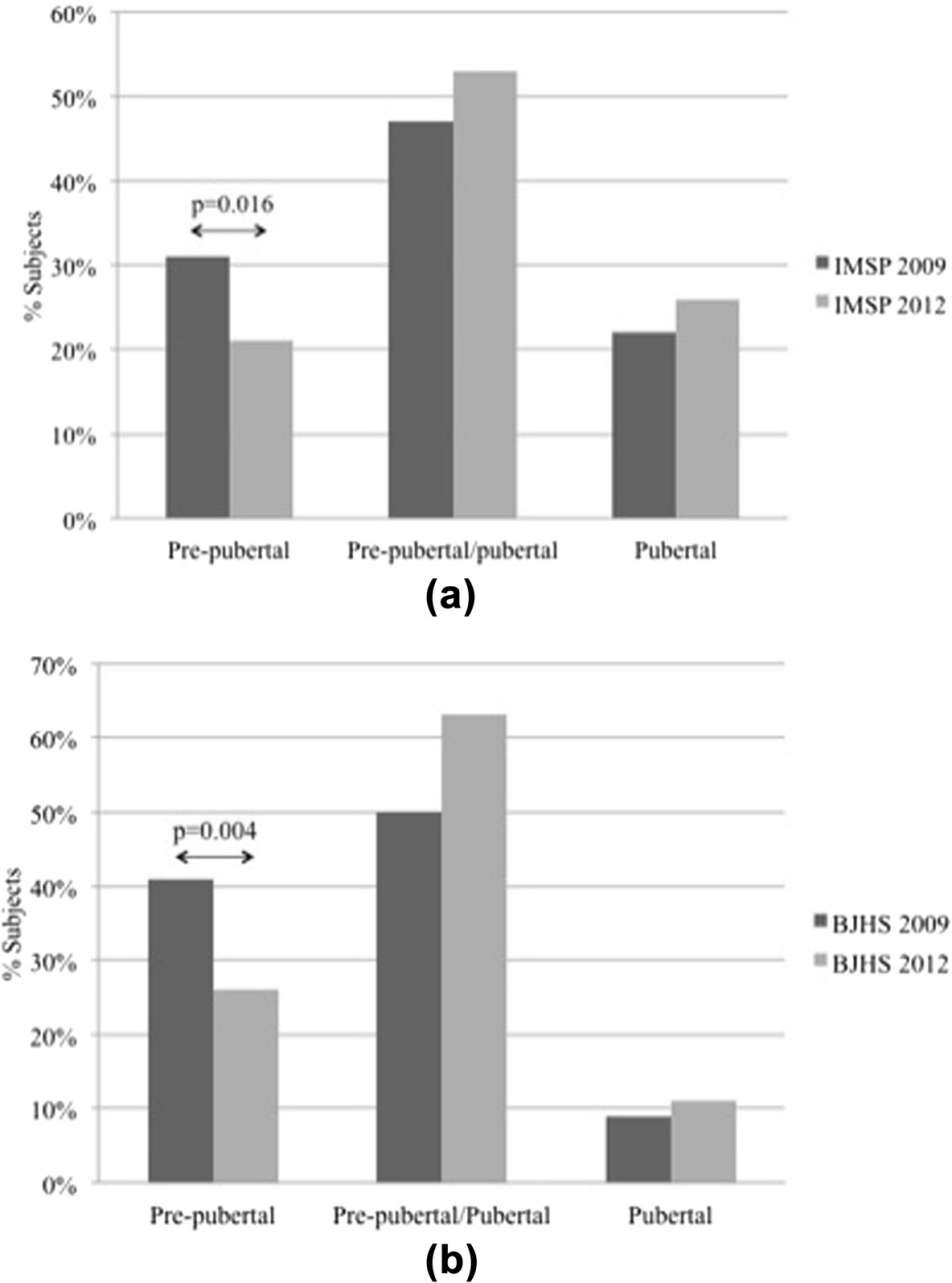
Change of Idiopathic Musculoskeletal Pain (**a**) and Benign Joint Hypermobility Syndrome (**b**) prevalence during the 2009–2012 period, according to the pubertal stage

By comparing the three groups we found that MSP persisted significantly more in pubertal (groups 2 and 3) rather than pre-pubertal subjects (group 1) (*p* = 0.022). In particular, pre-pubertal patients significantly recovered from BJHS (*p* = 0.004) and IMSP (*p* = 0.016) than the pubertal ones (Fig. 2). Moreover, BJHS persisted significantly more frequently in subjects that became pubertal during the 3 years period (group 2) (*p* = 0.015) than in the two other groups (data not shown). Gender did not influence the distribution of MSP since the gender distribution was not statistically significant among the three groups (*p* = 0.492).

Among the original 6 obese subjects, 4 were still obese at the last follow-up, and three of them still presented pain at the lower limbs (knees and ankles).

## Discussion

It is well known that puberty represents a risky period for the development of various rheumatologic conditions. For this reason, we have explored the role of puberty for the persistence of MSP [25]. Our study clearly shows that chronic non-inflammatory MSP persists across puberty, especially in females with BJHS.

This is the first prospective study in which musculoskeletal symptoms, rheumatologic evaluation and pubertal stage have been clinically evaluated by examining each patient during a 3 year interval time. In particular, to make the observation more reliable, we defined the pubertal status by validated clinical instruments, such as the Tanner tables and the Prader orchidometer.

Previous studies adopted a cross-sectional design [17, 26–32] while very few followed patients during time [11–16]. Indeed, these studies often involved large cohorts of patients but used survey questionnaires as instruments to collect data. Questionnaires are important for data collection and standardization, but certainly present some methodological limitations. A postal distributed questionnaire was probably characterized by a higher referral bias than a visit, and the compilation is not supervised: no explanation of questions could be given and no control to who compiles the questionnaire could be performed. Pediatric patients refer symptoms to their parents who fill the questionnaire, therefore the quality and the degree of reported pain is not always reliable. Indeed, the lack of physician-patient relationship implies the missing of objective measurements, which are essential for the correct evaluation of pain and become crucial when the credibility of the self-report measures may be questioned, as in the case of younger children [33]. A direct physical examination allows physician filtering parents’ information according to the real clinical status, avoiding, in this way, the over-and under-estimation of the symptoms [34]. Finally, clinical examination may allow detecting inflammatory or systemic diseases, such as arthritis or enthesitis, presenting as unspecific MSP at their onset.

Our results clearly show persistence of MSP during the teenage period since 54,3 % of the subjects, mostly pre-pubertal in 2009, were still symptomatic after 3 year follow-up. These data are consistent with several cross-sectional studies that showed an increased prevalence of MSP with age and a female predisposition [3, 26, 28, 32, 35].

Main reported symptoms were lower limbs arthralgia and myalgia. This anatomic localization of pain at weight bearing structures is probably due to the greater joint overloading that causes distraction of the capsule-ligamentous structures and muscle contractures. As expected, arthralgia was more frequent in patients with BJHS, which is mainly a joint disease. Myalgia was more common in subjects with IMSP, typically manifesting as cramping growing pains.

Interestingly, 47.2 % of subjects with previous IMSP and 61.8 % of those with previous BJHS continued to be symptomatic at the last follow. This means that both IMSP and BJHS heavily persist during time, with a slightly higher tendency to persist of BJHS than IMSP. Similar results have been obtained by the Authors in [36], who evaluated a cohort of patients with growing pains after a 5 year follow-up period.

More than one third of BJHS patients became asymptomatic but remained hypermobile. This suggests that hypermobility and pain have an independent natural history and are probably influenced by different factors.

Puberty plays an important role on the natural evolution of MSP since during the pre-pubertal period MSP tends to resolve. Conversely, pubertal subjects continue suffering from MSP to a greater extent than the pre-pubertal ones. A possible explanation could be that in this delicate period of life several physical, psychological, social and emotional components act as possible risk factors to develop or amplify the pain status [3, 13, 17].

This result is consistent with the clinical evidence of a higher prevalence of MSP among adolescents, previously reported by cross-sectional studies [3, 15, 28]. These studies, however, were carried out by questionnaires and, differently from ours, defined adolescence by age and not by clinical examination [12, 15–17].

Another new finding of our study is that BJHS represents the major determinant of MSP in puberty as compared with IMPS (p = 0,0015), with preference for the female gender. Puberty, therefore, seems not to influence BJHS as affected subjects remained both symptomatic and hypermobile after the 3 years observation period.

As for the role of obesity in determining MSP, only a descriptive analysis was performed because of the small number of cases. Its prevalence in our cohort is comparable with existing data [37]. Previous studies showed that obesity in children may be associated with MSP and may cause misalignments of the skeletal bones due to the excessive weight and joint capsule stretching [38, 39]. This was confirmed in our study since obese subjects referred symptoms mainly at the lower limbs where the joint overload is greater.

Possible limitations of the present study can be represented by the relative small size of the cohort and the possible selection bias at follow up since a proportion of patients who did not take part to the study might probably have become asymptomatic in the meantime. One of the strengths was that, conversely from previous reports [4, 16, 17], our study was not based on a survey through a questionnaire but all patients have been careful evaluated by physicians with good pediatric rheumatology training.

## Conclusion

In conclusion, while pre-pubertal subjects have a high probability of recovering from MSP, pubertal subjects are at high risk for suffering from MSP during early adulthood, particularly for females with BJHS. Our findings clearly suggest that female sex, BJHS and pubertal stage are important risk factors for persistence of MSP, and this is fundamental for a close monitoring of children and/or adolescents with MSP and for preventing the risk of suffering in adulthood.

Further studies are needed to evaluate the natural history of MSP towards adulthood and the potential links with the development of more aggressive conditions, such as osteoarthritis and rheumatoid arthritis.
